# Exploratory Detection of Nile Red-Positive Microparticles in Peripheral Blood Samples from Chronic Users of Nicotine Products Using Flow Cytometry

**DOI:** 10.3390/toxics14070611

**Published:** 2026-07-13

**Authors:** Justyna Śniadach, Aleksandra Starosz, Aleksandra Kicman, Kamila Jończyk, Anna Michalska-Falkowska, Damian Pachniewski, Sylwia Szymkowiak, Napoleon Waszkiewicz, Kamil Grubczak

**Affiliations:** 1 Department of Psychiatry, The Faculty of Medicine, Medical University of Bialystok, 15-272 Bialystok, Poland; psych@umb.edu.pl (A.K.); k.jonczyk9606@gmail.com (K.J.); napwas@wp.pl (N.W.); 2 Department of Regenerative Medicine and Immune Regulation, Medical University of Bialystok, Jerzego Waszyngtona 13, 15-269 Bialystok, Poland; medreg@umb.edu.pl (A.S.); damianp59@wp.pl (D.P.); 3 Biobank, Medical University of Bialystok, 15-269 Bialystok, Poland; biobank@umb.edu.pl; 4 Department of Genetics in Psychiatry, Poznan University of Medical Sciences, Rokietnicka 8, 60-806 Poznań, Poland; zgpsychiatria@ump.edu.pl

**Keywords:** Nile Red, microparticle, microplastic exposure, peripheral blood, nicotine use, human biomonitoring, flow cytometry, environmental exposure, tobacco products, nicotine dependence

## Abstract

Particles exhibiting fluorescence after Nile Red staining have been increasingly investigated in human biological samples in the context of microplastic exposure, but their clinical relevance remains unclear. In this study, Nile Red-positive microparticles were quantified in whole blood from chronic users of different nicotine-delivery systems and non-user controls. The study included 73 nicotine users, including conventional cigarette smokers, e-cigarette users, and heated tobacco product users, as well as an age- and sex-matched control group. Only participants without diagnosed chronic somatic disease were enrolled. Microparticles were quantified by flow cytometry after Nile Red staining. Associations with age, nicotine dependence assessed with the FTND and FTQ, motivation to quit smoking assessed with the Schneider Motivation Test, and selected hematological and biochemical parameters were also analyzed. In the overall analysis, the mean number of microparticles was higher in nicotine users than in controls, but the difference was not statistically significant (*p* = 0.38). In subgroup analyses, higher particle counts were observed in conventional cigarette smokers and heated tobacco product users compared with controls. No significant difference was found for e-cigarette users. Higher particle counts were also observed in older participants and in participants with greater nicotine dependence. Participants with higher particle counts more often showed stronger nicotine dependence and lower motivation to quit smoking. These results suggest that circulating Nile Red-positive microparticle counts may differ according to the type of nicotine product used and selected behavioral characteristics. However, the subgroup findings should be interpreted with caution due to the exploratory design and limited statistical power.

## 1. Introduction

Microplastics (MPs) are defined as plastic particles smaller than 5 mm, whereas nanoplastics (NPs) are particles smaller than 1 µm. These fractions differ in size, environmental behavior, and biological interactions [1,2]. In environmental and biological samples, microplastics are most commonly detected as polyethylene, polypropylene, polystyrene, and polyethylene terephthalate [3,4].

Microplastics are now recognized as widespread environmental contaminants present in air, water, and food products. Therefore, background exposure may occur in the general population, including individuals who do not use nicotine products. Their presence has also been demonstrated in the human body, which suggests distribution beyond the primary site of entry [5,6]. The detection of microplastics in internal tissues, including blood, indicates that translocation across biological barriers may occur [7,8]. Experimental and clinical studies also suggest that microplastics are not limited to primary exposure sites and may accumulate in organs such as the liver and lungs [9,10,11]. Due to their small size and large surface area, microplastics may interact with other environmental substances and have been associated with oxidative stress, inflammatory responses, and cytotoxic effects in experimental and preclinical studies [12,13,14].

Several exposure routes are considered relevant, including inhalation of airborne particles, gastrointestinal uptake through food and drinking water, and cutaneous absorption after direct skin contact [15,16]. Among these routes, inhalation may be particularly important in urban and indoor environments, where airborne microplastics are continuously present [1,3]. Airborne microplastics have already been reported in environmental air samples [17]. After inhalation, systemic circulation may potentially be reached through translocation. This route may be relevant for users of tobacco products and alternative nicotine delivery systems, as tobacco smoke and aerosols may contain combustion products, thermally degraded materials, and particles derived from polymer-based components [17,18].

In conventional cigarettes, cigarette filters may be an important source of polymer particle exposure [19]. Microplastics have also been detected in commercial e-liquids, which suggests that e-liquids themselves may represent an additional source of exposure among e-cigarette users [20]. Cigarette filters are mainly composed of cellulose acetate [21]. During smoking and inhalation, this material may fragment and generate microscale particles [19]. In heated tobacco products, potential sources of polymer particles may include filter-like elements, structural components of the device, and polymer-based materials exposed to heating. Polymer fibers released from filters may also persist in the environment, and their effects on ecological systems have been described [22,23]. However, systemic exposure to microplastics among users of different nicotine delivery systems remains poorly understood [18,24].

The biological relevance of microplastics detected in human tissues is still uncertain. Nanoplastics may be more biologically reactive than larger particles because of their size, surface properties, and potential for cellular internalization [2,25]. Inflammatory responses and oxidative stress have been reported in some studies, while other studies emphasize the need for further clinical data on systemic health effects [12,13]. Potential long-term health effects are still under evaluation [26].

A practical challenge in human biomonitoring studies is the detection of Nile Red-positive microparticles in complex biological matrices using methods applicable to larger clinical cohorts. Because Nile Red fluorescence is not specific to polymer composition, the detected microparticles should be interpreted as Nile Red-positive microparticles potentially associated with microplastic exposure rather than confirmed microplastics. Flow cytometry is a promising approach for rapid screening and characterization of microparticles by size and fluorescence properties [27]. Compared with more complex spectroscopic techniques, cytometry-based methods may offer a relatively accessible and high-throughput strategy for the exploratory assessment of microparticles in blood samples potentially associated with microplastic exposure, particularly in studies involving larger populations [4,28,29].

Tobacco smoke and aerosols generated by alternative nicotine delivery systems may represent a potential source of polymer-derived particles entering the human body. However, data on systemic exposure to microparticles potentially associated with microplastic exposure among users of different nicotine delivery systems remain scarce. Therefore, this study assessed circulating Nile Red-positive microparticles in peripheral blood by flow cytometry in users of conventional cigarettes, e-cigarettes, and heated tobacco products, and compared the results with non-user controls.

## 2. Materials and Methods

### 2.1. Study Groups

The study group included 73 users of nicotine products (mean age: 34.2 ± 11.3 years). This included regular cigarette smokers (*n* = 23), e-cigarette users (*n* = 25), and heated tobacco product users (*n* = 25). The control group consisted of 24 healthy individuals (mean age: 36.0 ± 10.7 years). Sex distribution was similar between groups (58% female, 42% male).

Participants aged 18–69 years were included. Regular use of the declared nicotine product for at least 12 months was required. Daily or near-daily use was confirmed based on medical history and self-report.

Nicotine dependence was assessed with the Fagerström Test for Nicotine Dependence. Clinical assessment was performed by a certified addiction psychotherapy specialist. Nicotine use history and dependence severity were evaluated during the clinical interview. Only one primary nicotine product was allowed. Participants with acute or chronic inflammatory conditions were excluded. These included active infection, autoimmune diseases, cardiovascular diseases, or cancer. Well-controlled arterial hypertension was allowed. Participants with recent infections (e.g., influenza, COVID-19, or common cold) were excluded.

Oral cavity status was assessed using a questionnaire. Participants with active dental inflammation, untreated caries, or recent tooth extraction were excluded. Participants did not report specific therapeutic or elimination diets. Regular high-intensity physical activity was not reported at the time of enrolment.

For nicotine-user groups, regular daily or near-daily use of one declared nicotine product for at least 12 months was required and confirmed based on medical history and self-report. Only one primary nicotine product was allowed. The control group included individuals with no current use of nicotine products. Nicotine dependence was assessed with the Fagerström Test for Nicotine Dependence. Clinical assessment was performed by a certified addiction psychotherapy specialist. Nicotine use history and dependence severity were evaluated during the clinical interview.

Detailed inclusion and exclusion criteria, together with controlled pre-analytical conditions applied during enrolment and biological sample collection, are provided in Supplementary Table S1.

The control group included clinically and laboratory-confirmed healthy subjects (n = 24). Matching by age and sex was performed. Individuals with inflammatory, autoimmune, or cancer conditions were excluded. Essential clinical and laboratory data are presented in Supplementary Materials (Supplementary Table S2).

The study protocol was approved by the Local Bioethics Committee of the Medical University of Bialystok (approval no. APK.002.95.2023). The study was conducted in accordance with the Declaration of Helsinki and local regulations. Written informed consent was obtained from all participants before enrollment. All data were anonymized before analysis.

### 2.2. Clinical Evaluation and Laboratory Diagnostics

Nicotine dependence was evaluated using two Fagerström-based tools and the Schneider motivation test. Severity of nicotine dependence was assessed using the Fagerström Test for Nicotine Dependence (FTND) [30]. This test evaluates physical nicotine dependence. Time to first cigarette after waking up and number of cigarettes smoked per day were included.

The Fagerström Tolerance Questionnaire (FTQ) was also used [31]. Behavioral and physiological components of nicotine dependence were assessed. Motivation to quit or reduce nicotine use was evaluated using the Schneider Motivation Test [32].

Clinical evaluation was performed using a structured medical interview. Assessment was carried out by a certified addiction psychotherapy specialist and a physician. Cumulative exposure history was additionally assessed during clinical interview and used to confirm chronic and regular nicotine-product use.

In conventional cigarette smokers, exposure was assessed using pack-years. In e-cigarette and heated tobacco product users, exposure duration reflected cumulative nicotine-use history rather than device-specific duration. Approximate daily use frequency and reported nicotine concentration were additionally recorded. Conventional cigarette smokers had a median cumulative exposure of 10.0 pack-years (IQR: 5.9–20.3; range: 1.1–40.0). E-cigarette users reported a median nicotine-use duration of 10.0 years (IQR: 6.0–15.5), with a median daily frequency above 20 uses/day. Heated tobacco product users reported a median nicotine-use duration of 11.0 years (IQR: 8.0–18.0), with a median daily frequency of 16–20 uses/day.

Oral cavity status was assessed using an author-developed questionnaire. The full author-developed oral health status questionnaire is provided as Supplementary File S1 Oral Health Status Questionnaire, including the English translation and the original Polish version used during data collection. Recent dental procedures were recorded. Caries was assessed. Gingival inflammation was assessed. Oral hygiene habits were also included. The questionnaire followed a standard oral health screening model [33].

Routine hematological and selected biochemical parameters were assessed in venous blood samples. Standardized pre-analytical procedures were applied. Morning blood collection was required. Fasting conditions were required. Alcohol consumption before sampling was not allowed. Intensive physical activity before sampling was not allowed.

Approximately 50 mL of venous blood was collected from each participant. Laboratory analyses were performed at the Population Research Center, Medical University of Białystok.

The following parameters were assessed: white blood cell count (WBC), differential leukocyte counts (lymphocytes, monocytes, granulocytes), red blood cell count (RBC), hemoglobin (HGB), hematocrit (HCT), mean corpuscular volume (MCV), mean corpuscular hemoglobin (MCH), mean corpuscular hemoglobin concentration (MCHC), red cell distribution width (RDW-CV, RDW-SD), platelet count (PLT), mean platelet volume (MPV), plateletcrit (PCT), platelet distribution width (PDW), platelet large-cell ratio (P-LCR), creatinine, estimated glomerular filtration rate (eGFR), sodium (Na^+^), potassium (K^+^), chloride (Cl^−^), urea, high-sensitivity C-reactive protein (hsCRP), aspartate aminotransferase (AST), alanine aminotransferase (ALT), glucose, and thyroid-stimulating hormone (TSH).

### 2.3. Assessment of Blood Microparticles

Whole blood samples anticoagulated with EDTA were collected from participants. The analytical procedure was based on the previously published protocol described by Salvia et al. [34], with modifications introduced for the present study. Spectroscopic techniques such as pyrolysis-GC/MS can identify polymers, but their use in biological samples is limited by complex sample preparation and lower analytical throughput [35,36]. Additional modifications of staining conditions and flow-cytometry settings were based on the work of Li et al. [37].

Venous blood samples were collected by trained medical personnel under standardized sterile conditions. For sample digestion, 20 µL of peripheral blood was mixed with 980 µL of 1% aqueous potassium hydroxide (KOH) solution (Sigma-Aldrich, St. Louis, MO, USA). After vortexing, samples were incubated on a heating block at 60 °C for 7 days.

After digestion, 100 µL of the processed sample was transferred to flow-cytometry tubes containing 900 µL of culture-grade ultrapure water. Samples were stained with 1 µg Nile Red (R&D Systems, Minneapolis, MN, USA) and incubated for 15 min with gentle shaking.

Flow-cytometry acquisition was performed using a FACSCalibur flow cytometer (BD Biosciences, San Jose, CA, USA). Data were analyzed using FlowJo software 10.8.2 (Tree Star Inc., Ashland, OR, USA). The gating strategy used for detection of Nile Red-positive microparticles in whole blood samples is presented in Supplementary Figure S1.

Particle-size gating was initially limited to events ≤2 µm using calibration beads (Flow Cytometry Size Calibration Kit, Thermo Fisher Scientific, Waltham, MA, USA). Nile-Red-positive events were identified using negative and positive control conditions. Negative controls included unstained blood samples, stained blood samples without added particles, procedural blanks, and instrument blanks. Positive controls consisted of polypropylene (PP) particles (100 µg) stained with Nile Red and tested in both blood matrix and buffer. Initial staining optimization was also performed using polystyrene (PS) particles, following previously published protocols [37]. Gating thresholds were established based on these control conditions. Data acquisition on two detectors was used as additional technical verification of the gating strategy.

To reduce potential external contamination, sample preparation was performed using laboratory consumables and reusable equipment rinsed with filtered ultrapure water before use. Standard contamination-control procedures were followed throughout the analytical process. Procedural blanks and instrument blanks were run in parallel with each batch to monitor background fluorescence and potential contamination. Reagents were prepared with filtered ultrapure water, and solutions were stored in glass containers whenever compatible with the analytical procedure. Sample tubes were kept covered during incubation. Cotton laboratory coats and powder-free gloves were worn throughout sample preparation, and work surfaces were cleaned before sample processing.

Based on these controls, gating thresholds were set to include only events corresponding to Nile Red-positive microparticles. This analytical approach enabled fluorescence-based detection of microparticles. However, polymer-specific identification of particles detected in patient blood samples was not possible with this method.

### 2.4. Biostatistical Analysis

Statistical processing of the study data was performed with the use of GraphPad Prism version 10.4.2 (GraphPad Prism Software Inc., San Diego, CA, USA). Basic differences between study groups were analyzed using a *t*-test or Mann–Whitney test, depending on the presence of a Gaussian distribution (assessed with the Shapiro–Wilk and Anderson–Darling tests). For correlation coefficient evaluation, Pearson or the nonparametric Spearman test was implemented. Risk analysis was performed with Fisher’s exact test, providing a relative risk (RR) value and 95% confidence interval in brackets. The statistical significance level was set at *p* < 0.05, with values indicated by exact values or asterisks: *—*p* < 0.05, **—*p* < 0.01, ***—*p* < 0.001, ****—*p* < 0.0001.

## 3. Results

### 3.1. Microparticles in Chronic Users of Selected Nicotine Products

**Figure 1:**
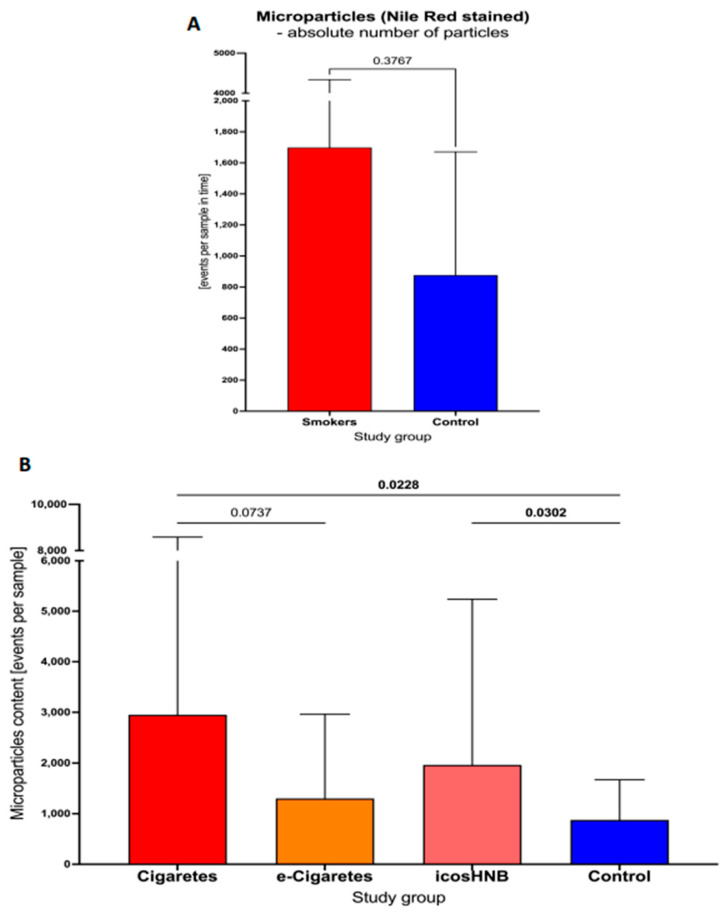
Levels of microparticle in whole blood assessed between smokers (nicotine users) and control group (**A**), including stratification into different nicotine products (**B**). Data presented as mean value and standard deviation, with significant differences indicated with exact *p* value.

The study group included 73 users of nicotine products, including regular cigarette smokers (*n* = 23), e-cigarette users (*n* = 25), and heat-not-burn tobacco product users (*n* = 25). The control group consisted of healthy non-users (*n* = 24). Despite higher mean values of Nile Red-positive microparticles in the peripheral blood of nicotine product users (*n* = 73), no significant differences were observed compared to the control group of healthy non-users (*n* = 24) (*p* = 0.3767) (Figure 1A).

Exposure history confirmed that all nicotine-user groups represented chronic and regular users rather than light or occasional exposure. We performed additional stratification of the smokers’ group for assessment of microparticles within different products groups. The cigarette-smoking group showed significantly higher levels of blood microparticles compared to the control group (*p* = 0.0228) and in subjects using IQOS-type heat-not-burn device products (*p* = 0.0302). No significant differences were observed between e-cigarette users and healthy non-users (Figure 1A).

### 3.2. Evaluation of Age Influence on Microparticle Levels in Peripheral Blood Among Smokers

**Figure 2:**
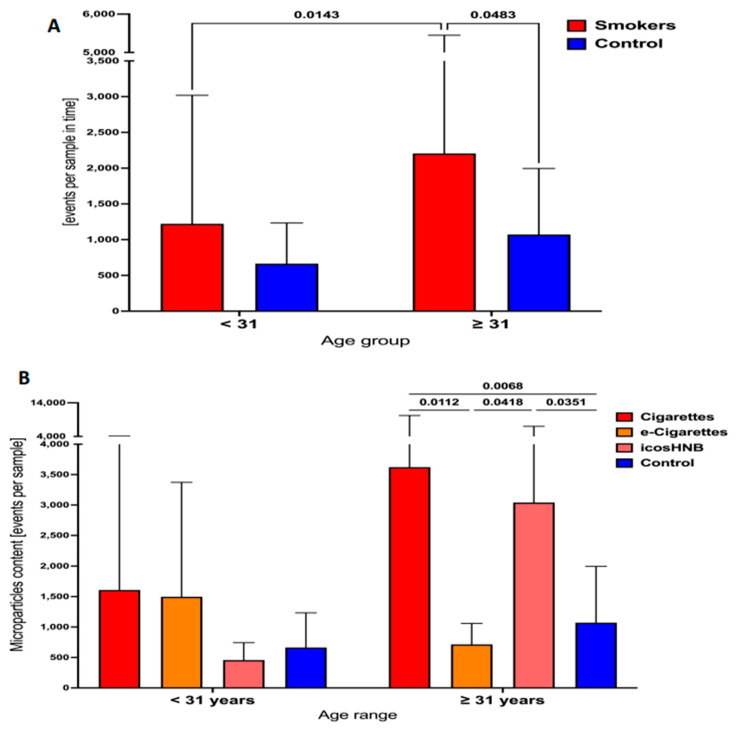
Assessment of circulating microparticles in the blood of smokers and the control group with age-based stratification (median age) (**A**), including additional product-type-based grouping (**B**). Data are presented as mean values ± standard deviation, with significant differences indicated by exact *p* values.

We further evaluated whether age was associated with microparticle levels in peripheral blood. Among younger smokers of selected nicotine products (under 31 years), no significant differences were observed, with or without additional stratification by product. However, in older subjects (31 years and older), significantly higher microparticle concentrations were observed not only compared with controls (*p* = 0.0483) but also compared with younger nicotine users (*p* = 0.0143) (Figure 2A).

Additional subgroup analysis of older subjects showed more pronounced significant differences when selected nicotine product users were compared with the non-user control group. Cigarette smokers (*p* = 0.0068) and icosHNB users (*p* = 0.0351) showed significantly higher values. Both groups were also significantly higher compared with e-cigarette users (*p* = 0.0112 and *p* = 0.0418, respectively) (Figure 2B).

### 3.3. Association Between Nicotine Dependence and Micoparticle Levels in Blood

**Figure 3:**
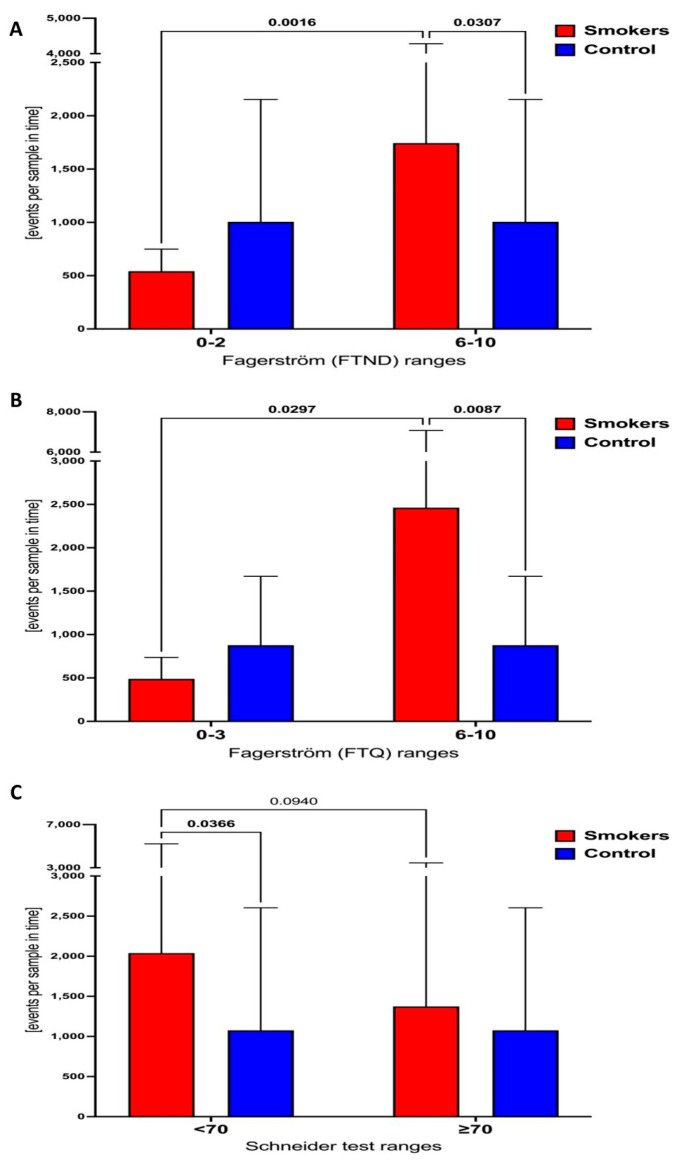
Differences in whole blood microparticle levels in smokers and control group including nicotine users’ stratification based on the results of Fagerström Test for Nicotine Dependence (FTND) (**A**) and Fagerström Tolerance Questionnaire (FTQ) (**B**), and Schneider (**C**) test. Data presented as mean value and standard deviation, with significant differences indicated with exact *p* value.

Considering the potentially longer duration of exposure in older subjects, the relationship between microparticle levels and nicotine dependence intensity was further evaluated. Dependence severity was assessed using FTND and FTQ. In FTND-based analysis, smokers with higher nicotine dependence (6–10 points) showed significantly higher microparticle levels compared with the control group (*p* = 0.0307). Similar findings were observed in FTQ-based stratification, where smokers with higher dependence also showed significantly higher values (*p* = 0.0087). No significant differences were observed between smokers and non-smokers in low-dependence subgroups (Figure 3A,B). In the Schneider test assessing motivation for smoking cessation, higher microparticle particle levels were observed in smokers compared with controls only among subjects with lower Schneider scores (<70), indicating lower motivation to quit smoking (*p* = 0.0366) (Figure 3C).

### 3.4. Correlations Between Blood Microparticle Levels and Selected Laboratory Parameters

In the next stage of analysis, associations between microparticle levels and selected laboratory parameters were evaluated. These included immune cell parameters (leukocytes), erythrocyte indices, platelet parameters, and selected biochemical markers. All analyses were further stratified by age (<31 years and ≥31 years).

**Figure 4:**
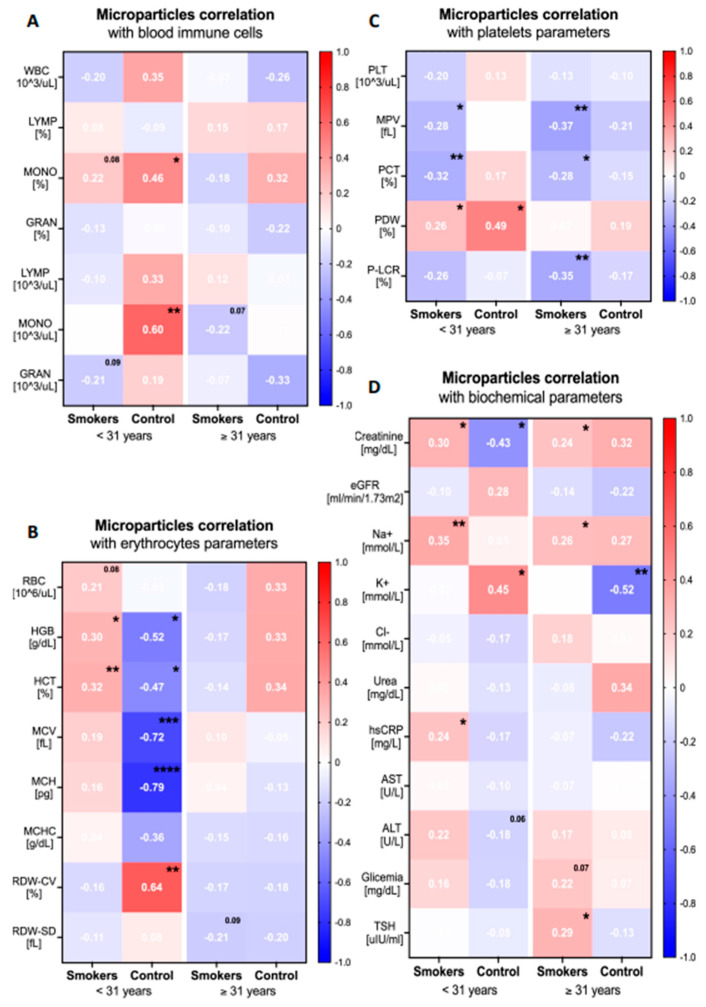
Association of microparticle content in whole blood, including study group (smokers or control) and age of subjects, with selected laboratory parameters related to: leukocytes (**A**), erythrocytes (**B**), platelets (**C**), biochemical parameters (**D**). Data presented as heat-maps of correlation coefficients, with significant correlations indicated with exact *p* value or asterisks. Asterisks indicate statistical significance: * *p* < 0.05; ** *p* < 0.01; *** *p* < 0.001; **** *p* < 0.0001.

In immune cell analysis, positive correlations between microparticle levels and monocyte-related parameters were observed mainly in the younger control group. These findings were not consistently present in smokers (Figure 4A).

In erythrocyte-related analysis, microparticle levels showed positive correlations with hemoglobin (HGB) and hematocrit (HCT) in younger smokers. In contrast, negative correlations were observed in the younger control group. Additional correlations were identified for mean corpuscular volume (MCV), mean corpuscular hemoglobin (MCH), and erythrocyte size variability parameters (RDW-CV) (Figure 4B).

In platelet-related analysis, mean platelet volume (MPV) and plateletcrit (PCT) showed negative correlations with microparticle levels in smokers. In younger subjects, platelet distribution width (PDW) showed positive correlations in both smokers and controls, although these associations were less evident in nicotine-user groups of older participants. In older smokers, a negative correlation was additionally observed for platelet large-cell ratio (P-LCR) (Figure 4C).

In biochemical analysis, positive correlations between microparticle levels and creatinine were observed in smokers, whereas the opposite association was observed in controls. In younger nicotine users, positive correlations were identified for sodium (Na^+^). In contrast, potassium (K^+^) showed opposite correlation directions in controls aged <31 years and ≥31 years. Additional positive associations were observed for high-sensitivity C-reactive protein (hsCRP) in younger smokers and thyroid-stimulating hormone (TSH) in smokers aged ≥31 years (Figure 4D).

### 3.5. Association Between Blood Microparticle Levels and Dependence- and Motivation-Related Test Results in Chronic Nicotine Users

**Figure 5:**
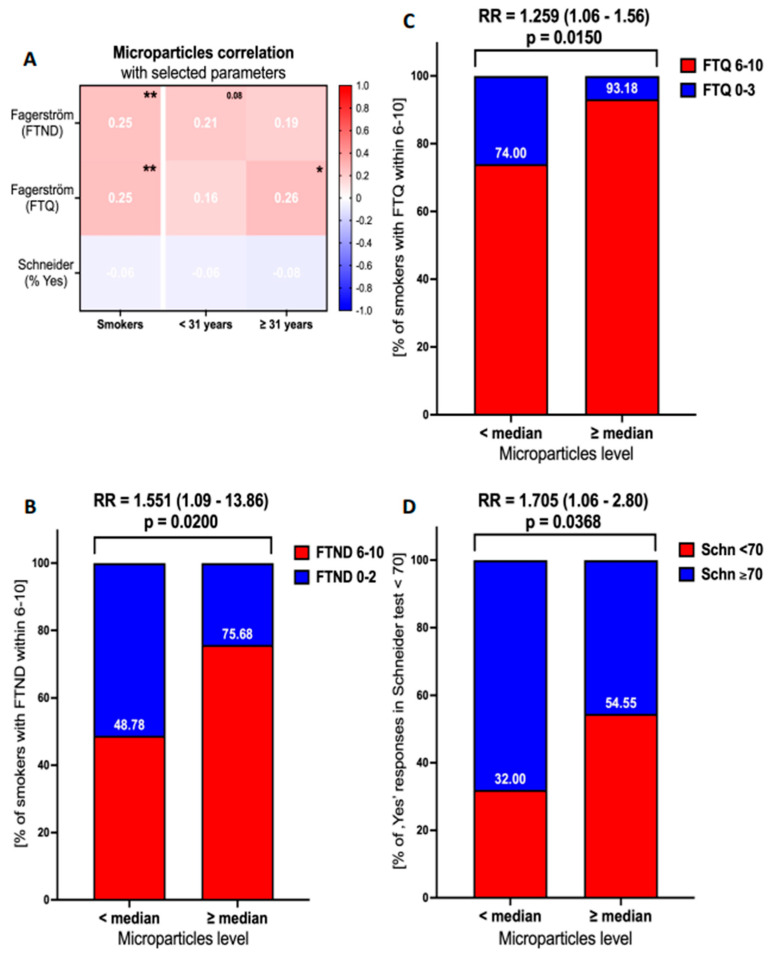
Associations between blood microparticle levels and dependence- and motivation-related test results in chronic nicotine users. (**A**) Correlation analysis of FTND, FTQ, and Schneider test results. (**B**,**C**) Relative risk (RR) analysis for higher FTND and FTQ scores according to blood microparticle level. (**D**) Relative risk (RR) analysis for lower motivation to smoking cessation (Schneider score < 70). Significant associations are indicated by exact *p*-values or asterisks. Asterisks indicate statistical significance: * *p* < 0.05; ** *p* < 0.01.

Associations between blood microparticle levels and dependence-related test results were further evaluated. Positive but relatively weak correlations were observed between microparticle levels and both Fagerström-based tests (FTND and FTQ). Age stratification did not have a major influence on these associations when subjects were divided at the median age of 31 years (Figure 5A).

Further analysis was performed to assess whether blood microparticle levels (below or above the group median) were associated with dependence severity and motivation for smoking cessation. Based on FTND results, smokers with higher microparticle levels showed a higher frequency of stronger nicotine dependence (6–10 points) compared with smokers with lower microparticle levels (75.68% vs. 48.78%; RR = 1.55; *p* = 0.0200). Similar findings were observed in FTQ-based analysis (93.18% vs. 74.00%; RR = 1.26; *p* = 0.0150) (Figure 5B,C).

In the Schneider test, smokers with higher blood microparticle levels were more frequently observed to have lower motivation for smoking cessation (<70% positive responses) compared with subjects below the median microparticle level (54.55% vs. 32.00%; RR = 1.71; *p* = 0.0368) (Figure 5D).

A summary of mean blood microparticle levels (±SD) across selected study groups, including stratification by age, nicotine product type, FTND, FTQ, and Schneider test results, is provided in Supplementary Table S3.

## 4. Discussion

The present analysis showed that chronic users of nicotine products had higher mean levels of Nile Red-positive microparticles in peripheral blood than non-users, although no significant difference was observed in the overall comparison. Additional stratification by nicotine product type revealed subgroup variability. Conventional cigarette smokers demonstrated significantly higher levels compared with controls, while a similar pattern was observed in users of heated tobacco products. No significant differences were identified between e-cigarette users and non-users. These findings should be interpreted with caution, particularly due to subgroup size and the exploratory character of fluorescence-based particle detection. Interpretation of exposure-related findings in users of conventional cigarettes, e-cigarettes, and heated tobacco products is challenging because these products differ in their design, aerosol generation, and biomarker profiles [38].

Previous biomonitoring studies have reported the presence of microplastic and nanoplastic particles in human blood and other biological matrices, indicating that environmental particles may enter the systemic circulation following inhalation or ingestion [7,8,26,39]. Animal studies have also shown tissue accumulation of microplastics and associated biomarker responses, supporting the biological plausibility of systemic effects [40]. Additional evidence has described biological interactions between micro- and nanoplastic particles and cells [41,42]. In the context of nicotine exposure, inhalation may represent one potential route of exposure to particulate material. Cigarette smoke contains a complex mixture of combustion-derived particles and other aerosol components. In addition, cigarette filters are primarily composed of cellulose acetate, which has been discussed as a possible source of particle release during smoking [13,24,43]. More broadly, the potential toxicity of microplastics and nanoplastics in mammalian systems has been described in previous experimental and review-based studies [44]. Microplastic particles may also act as carriers of chemical contaminants; however, the relevance of this mechanism to circulating particles in humans remains uncertain [45].

Age-stratified analysis suggested that differences in microparticles levels were more pronounced in older nicotine users. No significant differences were observed in subjects younger than 31 years. In contrast, participants aged 31 years and older showed higher particle levels compared with age-matched controls and younger nicotine users. Within this older subgroup, significantly higher levels were observed among cigarette smokers and heated tobacco users compared with non-users. Higher levels were also observed relative to e-cigarette users. One possible explanation may be longer cumulative exposure associated with age and prolonged nicotine product use. However, these findings should be interpreted cautiously and require confirmation in larger study groups.

Further analysis indicated that higher nicotine dependence scores were associated with higher levels of microparticles in peripheral blood. Smokers classified as highly dependent on FTND showed significantly higher particle levels than controls, with a similar pattern observed in the FTQ-based analysis. In contrast, lower dependence subgroups did not differ from non-users. In the Schneider smoking cessation motivation test, higher particle levels were observed mainly in smokers with lower motivation to quit smoking. These observations may reflect behavioral differences associated with nicotine-use intensity, including more frequent product use and repeated aerosol exposure. At the same time, dependence-related measures may also act as indirect markers of cumulative exposure. Therefore, these associations should be interpreted as observational and not causal [41,44,46].

Correlation analysis between blood microparticle levels and laboratory parameters was conducted with stratification by age and smoking status. Several associations differed across study groups.

In control subjects younger than 31 years, positive correlations were observed with monocyte-related parameters. Similar findings were not consistently identified in smokers. This may indicate differences between baseline physiological patterns and nicotine-related exposure effects, although no direct conclusions can be drawn.

Erythrocyte-related parameters were also analyzed. No clear association was observed between erythrocyte count and blood microparticle levels. Among younger smokers, positive correlations were observed between hemoglobin and hematocrit. In younger controls, inverse correlations were observed. Additional associations were observed for erythrocyte-related indices, including mean corpuscular volume, mean corpuscular hemoglobin, and red blood cell distribution width. These patterns were not reproduced consistently across all study groups. Importantly, many of the observed correlations were not reproduced consistently across age groups and smoking categories, and in some cases, opposite directions of association were identified. These findings limit biological interpretation and suggest that the observed relationships may reflect statistical variability, subgroup-specific effects, limited sample size after stratification, or residual confounding rather than true biological mechanisms. Therefore, these associations should be considered exploratory and hypothesis-generating until confirmed in larger, independently powered studies.

Platelet-related indices showed additional associations. In smokers, mean platelet volume and plateletcrit showed negative correlations with microparticle levels. Platelet distribution width showed positive correlations in younger smokers and controls. In older smokers, an additional negative association was observed for platelet large-cell ratio. Platelet-related alterations have previously been discussed in experimental models of particulate exposure, particularly in the context of inflammatory and oxidative processes. Similar systemic effects were reported after inhalation exposure to airborne particulate matter [47]. Changes in platelet activation and inflammatory signaling were observed in experimental models [48]. Experimental studies have also shown that microplastic particles may affect oxidative balance and inflammatory regulation [49]. Broader toxicological and epidemiological evidence on airborne particulate matter and e-cigarette aerosols also links particle size, particle composition, and inhaled aerosol exposure with oxidative damage, cardiovascular mechanisms, and inflammatory responses [50,51,52]. However, the clinical relevance of these findings in humans remains unclear. It is unknown whether circulating Nile Red-positive microparticles are associated with measurable metabolic, inflammatory, or platelet-related effects in humans.

Further analysis showed weak positive correlations between blood microparticle levels and both Fagerström-based tests. Age stratification did not markedly change these findings. When subjects were divided according to particle levels above and below the group median, smokers with higher microparticle levels were more likely to be classified with high or very high nicotine dependence in both FTND- and FTQ-based analysis. A similar relationship was observed in subjects with lower motivation to smoking cessation according to the Schneider test.

Therefore, MLP levels should be interpreted as potential markers associated with nicotine-use intensity and exposure patterns rather than determinants of nicotine dependence. Similar relationships between behavior and environmental exposure were described previously [13].

Several limitations must be noted. The study had an exploratory design, and subgroup sizes were limited. Flow cytometry combined with Nile Red staining was used as a screening method. This approach was rapid and relatively accessible for blood-sample analysis. However, polymer-specific identification was not possible. More advanced methods, including Raman spectroscopy, μFTIR, and pyrolysis-GC/MS, provide more detailed chemical characterization. Direct comparisons between nicotine-user groups should also be interpreted with caution. Exposure patterns differed between cigarette smokers, e-cigarette users, and heated tobacco users. Pack-years, duration of use, daily frequency, and aerosol exposure were not fully comparable between groups. This was particularly relevant for e-cigarette and heated tobacco users. In addition, interpretation of cumulative nicotine history may have been influenced by prior conventional cigarette smoking and switching between nicotine-product types, making the distinction between device-specific and overall exposure more challenging.

## 5. Conclusions

Nile Red-positive microparticles were detected in peripheral blood samples collected from both chronic nicotine users and non-users. In subgroup analyses, higher levels were observed in cigarette smokers and heated tobacco users. No significant difference was found when the entire nicotine-user group was compared with controls.

Higher blood microparticle levels were related to stronger nicotine dependence in selected analyses. A similar pattern was seen for lower motivation to smoking cessation in the Schneider test. This may reflect differences in smoking behavior and inhalation-related exposure.

Flow cytometry combined with Nile Red staining was used for fluorescence-based detection of microparticles in blood. This approach enabled rapid, quantitative assessment of particle-like fluorescent events. It did not allow polymer-specific identification. For this reason, the results should be interpreted carefully.

## Data Availability

The original contributions presented in this study are included in the article/Supplementary Material. Further inquiries can be directed to the corresponding authors.

## Supplementary Materials

The following supporting information can be downloaded at https://www.mdpi.com/article/10.3390/toxics14070611/s1, Table S1: Inclusion and exclusion criteria and controlled pre-analytical conditions applied during participant enrolment and biological sample collection; Table S2: Clinical and laboratory characteristics of the study groups. Data presented as mean values with standard deviation, and *p* value of differences between groups; Table S3: Mean values of blood microplastics (with standard deviation - ± SD) in selected study groups including stratification based on age, nicotine product type, results of Fagerström (FTND), Fagerström (FTQ), and Schneider tests; Figure S1; Figure S2; Supplementary File S1: Oral Health Status Questionnaire.

## Abbreviations

AST	Aspartate aminotransferase
ALT	Alanine aminotransferase
Cl^−^	Chloride
EDTA	Ethylenediaminetetraacetic acid
eGFR	Estimated glomerular filtration rate
FTND	Fagerström Test for Nicotine Dependence
FTQ	Fagerström Tolerance Questionnaire
HCT	Hematocrit
HGB	Hemoglobin
HNB	Heat-not-burn
hsCRP	High-sensitivity C-reactive protein
K^+^	Potassium
KOH	Potassium hydroxide
MCV	Mean corpuscular volume
MCH	Mean corpuscular hemoglobin
MCHC	Mean corpuscular hemoglobin concentration
MP	Microplastics
MPV	Mean platelet volume
Na^+^	Sodium
NPs	Nanoplastics
PCT	Plateletcrit
PDW	Platelet distribution width
PLT	Platelet count
PP	Polypropylene
PS	Polystyrene
P-LCR	Platelet large-cell ratio
RBC	Red blood cell count
RDW-CV	Red cell distribution width-coefficient of variation
RDW-SD	Red cell distribution width-standard deviation
RR	Relative risk
TSH	Thyroid-stimulating hormone
WBC	White blood cell count
