# Supplementary material for: Exploratory Detection of Nile Red-Positive Microparticles in Peripheral Blood Samples from Chronic Users of Nicotine Products Using Flow Cytometry
Source: Toxics. 2026 Jul 13;14(7):611. doi: 10.3390/toxics14070611 (PMC13419147; doi:10.3390/toxics14070611)
Supplement: Supplementary file 1 [file toxics-14-00611-s001.zip › Supplementary Figure S2.pdf]

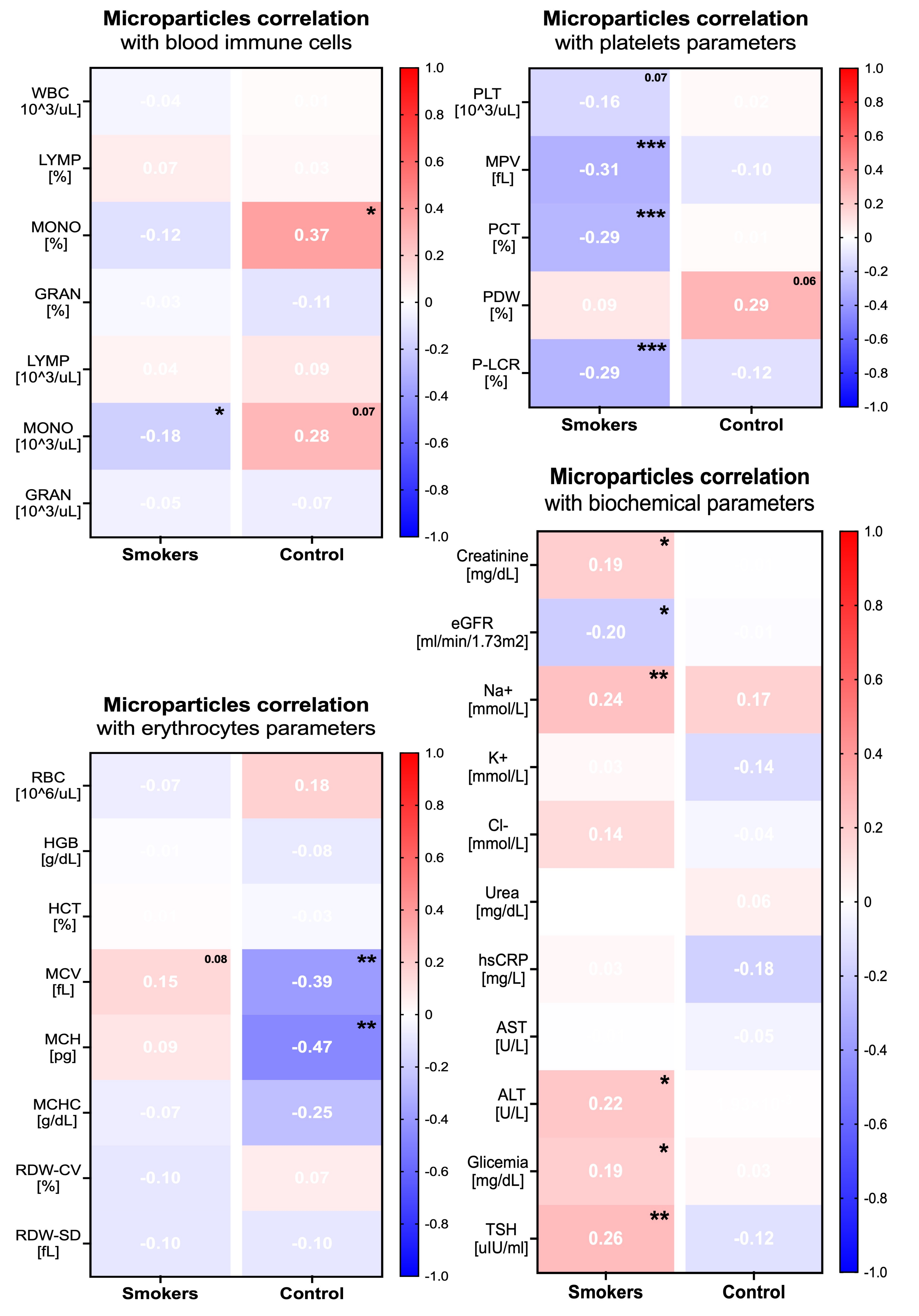
Microparticles correlation
with blood immune cells
WBC 10^3/uL]
LYMP [%]
MONO [%]
GRAN [%]
LYMP [10^3/uL]
MONO [10^3/uL]
GRAN [10^3/uL]
-0.04
0.07
0.03
-0.12
0.37
*
-0.03
-0.11
0.04
0.09
-0.18
*
0.28
0.07
-0.05
-0.07
Smokers
Control
1.0
0.8
0.6
0.4
0.2
0
-0.2
-0.4
-0.6
-0.8
-1.0
Microparticles correlation
with platelets parameters
PLT [10^3/uL]
MPV [fL]
PCT [%]
PDW [%]
P-LCR [%]
-0.16
0.07
0.02
-0.31
***
-0.10
-0.29
***
0.09
0.29
0.06
-0.29
***
-0.12
Smokers
Control
Microparticles correlation
with erythrocytes parameters
RBC [10^6/uL]
HGB [g/dL]
HCT [%]
MCV [fL]
MCH [pg]
MCHC [g/dL]
RDW-CV [%]
RDW-SD [fL]
-0.07
0.18
-0.08
-0.03
0.15
0.08
-0.39
**
0.09
-0.47
**
-0.07
-0.25
-0.10
0.07
-0.10
-0.10
Smokers
Control
Microparticles correlation
with biochemical parameters
Creatinine [mg/dL]
eGFR [ml/min/1.73m2]
Na+ [mmol/L]
K+ [mmol/L]
Cl- [mmol/L]
Urea [mg/dL]
hsCRP [mg/L]
AST [U/L]
ALT [U/L]
Glicemia [mg/dL]
TSH [uIU/ml]
0.19
*
-0.20
*
0.24
**
0.17
-0.14
0.14
-0.04
0.06
-0.18
-0.05
0.22
*
0.19
*
0.26
**
-0.12
Smokers
Control
