# Supplementary material for: Exploratory Detection of Nile Red-Positive Microparticles in Peripheral Blood Samples from Chronic Users of Nicotine Products Using Flow Cytometry
Source: Toxics. 2026 Jul 13;14(7):611. doi: 10.3390/toxics14070611 (PMC13419147; doi:10.3390/toxics14070611)
Supplement: Supplementary file 1 [file toxics-14-00611-s001.zip › Supplementary File S1 Oral Health Status Questionnaire.pdf]

Name and surname: .... **Date:** ....

Oral Health Status Questionnaire. **Please select the appropriate answer.**

1. **When was your last visit to a dentist/dental hygienist?**
   a) Within the last 3 months
   b) Within the last 6 months
   c) Within the last year
   d) Within the last 2 years or more
   e) I do not remember
2. **Do you think that you currently have dental caries [cavities] and/or pathological changes in the oral cavity?**
   a) No, I do not think so
   b) Yes, I have single cavities (1–3) in the oral cavity
   c) Yes, I have dental caries affecting more than 3 teeth and tooth pain
   d) I do not know
3. **Do you experience pain when eating hot/cold foods?**
   a) Yes
   b) No
   c) I do not know
4. **Have you noticed painful or bleeding gums in the last 3 months?**
   a) Yes
   b) No
   c) I do not know
5. **Has any dentist/dental hygienist ever told you that you have deep periodontal pockets, i.e., receding gums?**
   a) Yes
   b) No
   c) I do not remember
6. **Has a dentist or dental hygienist ever told you that you suffer from periodontal disease or gum disease?**
   a) Yes
   b) No
   c) I do not remember
7. **Have you had any dental procedures performed in the last 3 months, such as tooth extraction [removal], implant placement, root canal treatment, or other procedures?**
   a) Yes
   b) No
   c) I do not remember
8. **How many times a day do you brush your teeth?**
   a) I sometimes forget to brush my teeth every day
   b) Once a day
   c) Twice a day
   d) Three times a day or more often
9. **During your daily oral hygiene routine, do you use dental floss and/or mouthwash?**
   a) Yes, I use dental floss
   b) Yes, I use mouthwash
   c) Yes, I use both dental floss and mouthwash
   d) I use these products occasionally
   e) No, I do not use these products

Imię i nazwisko ………………………………………………… Data …………………

Kwestionariusz dotyczący stanu zdrowia jamy ustnej. Zakreśl właściwą odpowiedź.

1. Kiedy ostatni raz byłeś u dentysty/higienistki stomatologicznej?
   a) W przeciągu ostatnich 3 miesięcy
   b) W przeciągu ostatnich 6 miesięcy
   c) W przeciągu ostatniego roku
   d) W przeciągu ostatnich 2 lat i więcej
   e) Nie pamiętam
2. Czy uważasz, że masz teraz próchnicę [ubytki] lub zmiany chorobowe w jamie ustnej?
   a) Nie, nie sądzę
   b) Tak, mam pojedyncze ubytki (1-3) w jamie ustnej
   c) Tak, mam próchnicę (więcej niż 3 zębów)i ból/e zęba/ów
   d) Nie wiem
3. Czy odczuwasz ból podczas jedzenia gorących/zimnych pokarmów?
   a) Tak
   b) Nie
   c)Nie wiem
4. Czy zaobserwowałeś u siebie w ciągu ostatnich 3 miesięcy bolesne lub krwawiące dziąsła?
   a) Tak
   b Nie
   c) Nie wiem
5. Czy jakikolwiek dentysta/higienistka mówił/a Ci kiedykolwiek, że masz głębokie kieszonki tj. cofające się dziąsła?
   a) Tak
   b) Nie
   c) Nie pamiętam
6. Czy dentysta lub higienistka stomatologiczna kiedykolwiek powiedział/a Ci, że cierpisz na chorobę przyzębia lub dziąseł?
   a) Tak
   b) Nie
   c) Nie pamiętam
7. Czy w ciągu ostatnich 3 miesięcy miałeś wykonywane zabiegi dentystyczne takie jak ekstrakcja [usuwanie] zęba, wstawianie implantu, kanałowe leczenie zęba i inne?
   a) Tak
   b) Nie
   c) Nie pamiętam
8. Ile razy dziennie myjesz zęby?
   a) Zdarza mi się zapominać o codziennym myciu zębów
   b) Raz dziennie
   c) Dwa razy dziennie
   d) 3 razy więcej i częściej
9. Czy podczas codziennej higieny jamy ustnej używasz nici dentystycznej i/lub płynu do płukania jamy ustnej?
   a) Tak, używam nici dentystycznej
   b) Tak, używam płynu do płukania jamy ustnej
   c) Tak, używam zarówno nici dentystycznych jak i płynu do płukania jamy ustnej
   d) Używam tych produktów sporadycznie
   e) Nie, nie używam tych produktów
