# Supplementary material for: Exploratory Detection of Nile Red-Positive Microparticles in Peripheral Blood Samples from Chronic Users of Nicotine Products Using Flow Cytometry
Source: Toxics. 2026 Jul 13;14(7):611. doi: 10.3390/toxics14070611 (PMC13419147; doi:10.3390/toxics14070611)
Supplement: Supplementary file 1 [file toxics-14-00611-s001.zip › Supplementary Table S1.pdf]

Supplementary Table S1. Inclusion and exclusion criteria and controlled pre-analytical conditions applied during participant enrolment and biological sample collection.

Category	Criterion or controlled condition	Applies to / clarification
	**INCLUSION CRITERIA**	
Inclusion criteria	Age 18–69 years	All participants
Inclusion criteria	Regular daily or near-daily use of one declared nicotine product for at least 12 months	Nicotine-user groups
Inclusion criteria	Absence of nicotine product use	Control group
Inclusion criteria	Completion of standardized clinical interview, questionnaires, and full clinical qualification	All participants
Inclusion criteria	Biological sample collection according to the study protocol	All participants
Inclusion criteria	Written informed consent obtained before enrolment	All participants
Inclusion criteria	Good general health status at enrolment	All participants
	**EXCLUSION CRITERIA**	
Exclusion criteria	Use of more than one nicotine product	Nicotine-user groups
Exclusion criteria	Occasional or non-regular nicotine product use	Nicotine-user groups
Exclusion criteria	Chronic diseases, autoimmune diseases, cancer, or other relevant medical conditions potentially affecting inflammatory, clinical, or laboratory outcomes	All participants
Exclusion criteria	Active infection or recent infection, including influenza, COVID-19, or common cold	All participants
Exclusion criteria	Acute inflammatory conditions or other active inflammatory processes	All participants
Exclusion criteria	Oral cavity abnormalities potentially affecting clinical evaluation or inflammatory markers	Including gingival inflammation, active caries, diseased teeth, recent dental procedures, or tooth extraction
Exclusion criteria	Menstruation at the planned time of biological sample collection	Sampling was postponed
Exclusion criteria	Abnormal toxicological or clinical findings resulting in exclusion from analysis	Including elevated THC
Exclusion criteria	Incomplete clinical, questionnaire-based, or qualification data	All participants

**Category**	**Criterion or controlled condition**	**Applies to / clarification**
	**PRE-ANALYTICAL AND CONTROLLED FACTORS**	
Pre-analytical factors	Standardized morning biological sample collection	Fixed collection window
Pre-analytical factors	Fasting conditions before sampling	All participants
Pre-analytical factors	Water permitted before sampling	All participants
Pre-analytical factors	No smoking or nicotine product use for at least 1 hour before sampling	Nicotine-user groups
Pre-analytical factors	No coffee intake before sampling	All participants
Pre-analytical factors	Exposure history recorded during clinical interview	Pack-years, years of product use, and approximate daily frequency
Pre-analytical factors	Dental interview and oral cavity assessment	All participants
