# Supplementary material for: Exploratory Detection of Nile Red-Positive Microparticles in Peripheral Blood Samples from Chronic Users of Nicotine Products Using Flow Cytometry
Source: Toxics. 2026 Jul 13;14(7):611. doi: 10.3390/toxics14070611 (PMC13419147; doi:10.3390/toxics14070611)
Supplement: Supplementary file 1 [file toxics-14-00611-s001.zip › Supplementary Table S2.pdf]

	Smokers	Control	p value
Age	34.2 (± 11.3)	36.0 (± 10.7)	0.5209
Sex [female/male]	42 (58%) / 31 (42%)	14 (58%) / 10 (42%)	0.9999
WBC [10^3/ul]	6.33 (± 1.64)	5.33 (± 1.06)	0.7295
Lymphocytes [%]	31.46 (± 7.01)	36.31 (± 7.54)	0.0929
Monocytes [%]	7.93 (± 1.95)	7.26 (± 1.63)	0.8174
Granulocytes [%]	60.56 (± 7.41)	56.41 (± 7.40)	0.1511
Lymphocytes [10^3/ul]	1.95 (± 0.57)	1.91 (± 0.40)	0.9882
Monocytes [10^3/ul]	0.49 (± 0.15)	0.39 (± 0.12)	0.9702
Granulocytes [10^3/ul]	3.89 (± 1.31)	3.04 (± 0.82)	0.7673
RBC [10^6/ul]	4.68 (± 0.40)	4.56 (± 0.40)	0.9660
HGB [g/dl]	14.07 (± 1.20)	13.33 (± 1.27)	0.7973
HCT [%]	41.76 (± 3.33)	39.68 (± 3.71)	0.4706
MCV [fL]	89.29 (± 4.19)	87.00 (± 3.78)	0.4288
MCH [pg]	30.08 (± 1.58)	29.23 (± 1.39)	0.7700
MCHC [g/dl]	33.67 (± 0.81)	33.60 (± 0.67)	0.9785
RDW-CV [%]	12.45 (± 0.95)	12.95 (± 1.17)	0.8624
RDW-SD [fL]	40.24 (± 2.68)	38.98 (± 2.80)	0.6626
PLT [10^3/ul]	241.3 (± 59.3)	247.9 (± 62.18)	0.0237
MPV [fL]	9.79 (± 1.29)	9.07 (± 1.09)	0.8007
PCT [%]	0.24 (± 0.06)	0.22 (± 0.05)	0.9967
PDW [%]	13.29 (± 1.81)	14.13 (± 1.50)	0.7720
P-LCR [%]	22.99 (± 10.13)	15.92 (± 7.40)	0.0144
Creatinine [mg/dl]	0.87 (± 0.15)	0.88 (± 0.15)	0.9996
eGFR [ml/min/1.73m2]	102.7 (± 14.63)	100.1 (± 13.26)	0.3618
Na+ [mmol/L]	139.5 (± 1.82)	139.6 (± 1.59)	0.9885
K+ [mmol/L]	4.51 (± 0.32)	4.53 (± 0.39)	0.9967
Cl- [mmol/L]	103.0 (± 1.92)	102.8 (± 1.82)	0.9612
Urea [mg/dl]	27.66 (± 6.61)	27.09 (± 6.68)	0.8393
hsCRP [mg/dl]	1.34 (± 2.50)	0.78 (± 1.12)	0.8445
AST [U/L]	26.47 (± 10.86)	27.78 (± 11.15)	0.6466
ALT [U/L]	26.08 (± 15.69)	23.87 (± 12.49)	0.4362
Glicemia [mg/dl]	92.30 (± 9.02)	95.08 (± 11.96)	0.3270
TSH [uIU/ml]	2.12 (± 2.01)	1.84 (± 0.85)	0.9199

Table S2. Clinical and laboratory characteristics of the study groups. Data presented as mean values with standard deviation, and p value of differences between groups.
