# Supplementary material for: Exploratory Detection of Nile Red-Positive Microparticles in Peripheral Blood Samples from Chronic Users of Nicotine Products Using Flow Cytometry
Source: Toxics. 2026 Jul 13;14(7):611. doi: 10.3390/toxics14070611 (PMC13419147; doi:10.3390/toxics14070611)
Supplement: Supplementary file 1 [file toxics-14-00611-s001.zip › Supplementary Table S3.pdf]

Microplastics mean values (with ± SD) in selected groups		
**Nicotine users versus control**		
Smokers	1699 (± 2640)	
Control	877 (± 794)	
**Nicotine products groups versus control**		
Cigarettes	2951 (± 5634)	
e-Cigarettes	1301 (± 1663)	
icosHNB	1964 (± 3273)	
Control	877 (± 794)	
**Age influence on microplastics in selected groups**		
	**< 31 years**	**≥ 31 years**
**Nicotine users versus control**		
Smokers	1222 (± 1797)	2204 (± 3246)
Control	664 (± 569)	1071 (± 924)
**Nicotine products groups versus control**		
Cigarettes	1607 (± 2507)	3622 (± 6616)
e-Cigarettes	1497 (± 1876)	714 (± 345)
icosHNB	457 (± 285)	3041 (± 3963)
Control	664 (± 569)	1071 (± 924)
**Fagerström (FTND) and microplastics in smokers versus control**		
	**0-2 FTND**	**6-10 FTND**
Smokers	542 (± 207)	1744 (± 2537)
Control	1005 (±1148)	
**Fagerström (FTQ) and microplastics in smokers versus control**		
	**0-3 FTQ**	**6-10 FTQ**
Smokers	489 (± 248)	2462 (± 4608)
Control	877 (± 794)	
**Schneider score and microplastics in smokers versus control**		
	**< 70**	**≥ 70**
Smokers	2039 (± 3179)	1374 (± 2083)
Control	1077 (± 1527)	

Supplementary Table S3. Mean values of blood microplastics (with standard deviation - ± SD) in selected study groups including stratification based on age, nicotine product type, results of Fagerström (FTND), Fagerström (FTQ), and Schneider tests.
