# Supplementary figures and images for: Exploratory Detection of Nile Red-Positive Microparticles in Peripheral Blood Samples from Chronic Users of Nicotine Products Using Flow Cytometry
Source: Toxics. 2026 Jul 13;14(7):611. doi: 10.3390/toxics14070611 (PMC13419147; doi:10.3390/toxics14070611)

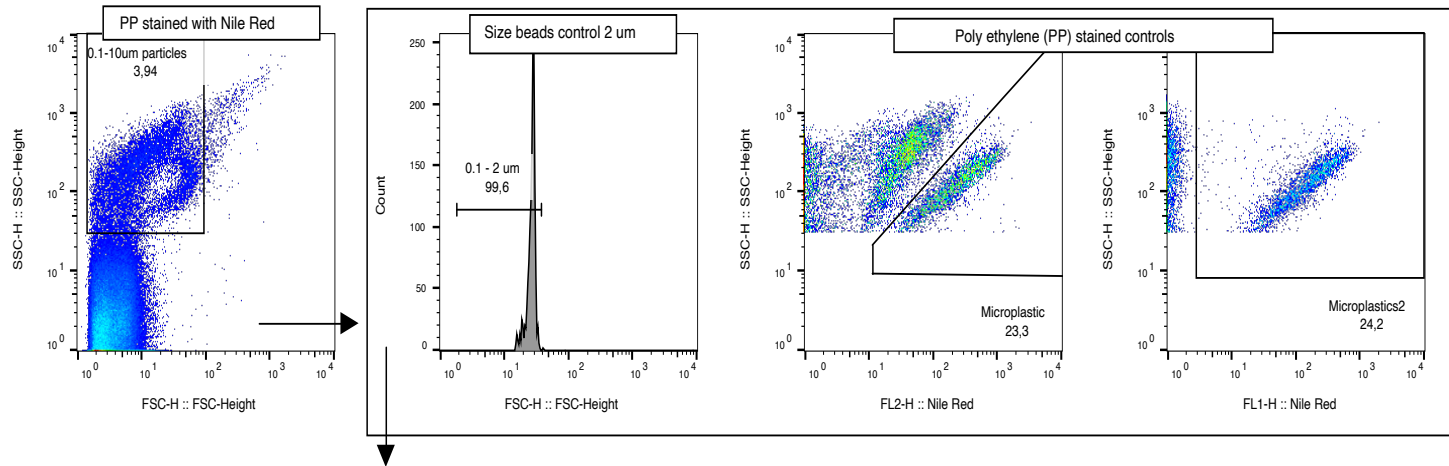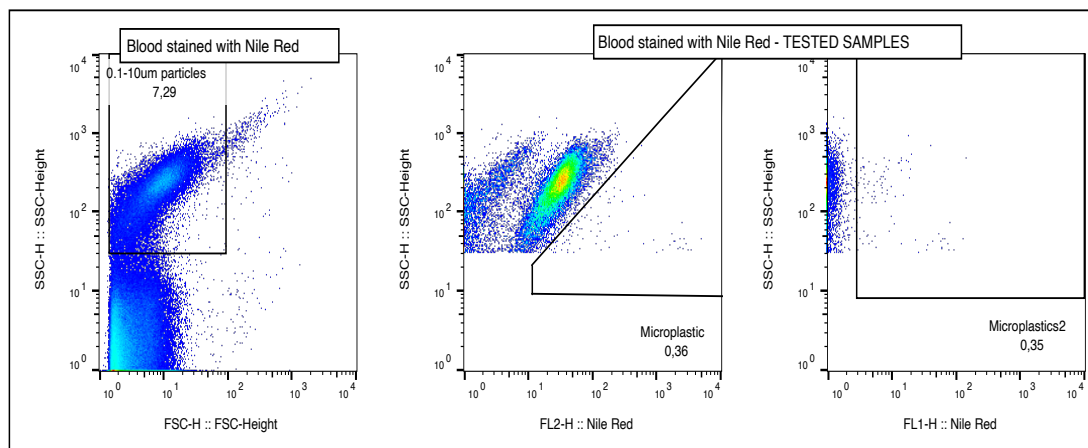

Supplement: Supplementary file 1 [file toxics-14-00611-s001.zip › Supplementary Figure S1.pdf]
